# ESBL-Producing *Escherichia* *coli* Carrying CTX-M Genes Circulating among Livestock, Dogs, and Wild Mammals in Small-Scale Farms of Central Chile

**DOI:** 10.3390/antibiotics10050510

**Published:** 2021-04-30

**Authors:** Julio A. Benavides, Marília Salgado-Caxito, Andrés Opazo-Capurro, Paulina González Muñoz, Ana Piñeiro, Macarena Otto Medina, Lina Rivas, Jose Munita, Javier Millán

**Affiliations:** 1 Departamento de Ecología y Biodiversidad, Facultad de Ciencias de la Vida, Universidad Andrés Bello, Santiago 8320000, Chile; mottomed@gmail.com (M.O.M.); syngamustrachea@hotmail.com (J.M.); 2 Centro de Investigación para la Sustentabilidad, Facultad de Ciencias de la Vida, Universidad Andrés Bello, Santiago 8320000, Chile; 3 Millennium Initiative for Collaborative Research on Bacterial Resistance (MICROB-R), Santiago 7550000, Chile; mariliasalgadovet@gmail.com (M.S.-C.); andopazo@udec.cl (A.O.-C.); paulinagonzalez@udec.cl (P.G.M.); linarivas@udd.cl (L.R.); munita.jm@gmail.com (J.M.); 4 School of Veterinary Medicine, Pontificia Universidad Católica de Chile, Santiago 7820244, Chile; 5 Departamento de Microbiología, Facultad de Ciencias Biológicas, Universidad de Concepción, Concepción 4070386, Chile; 6 Departamento de Ciencias Biológicas y Químicas, Facultad de Medicina y Ciencia, Universidad San Sebastián, Concepción 4030000, Chile; 7 Escuela de Medicina Veterinaria, Facultad de Ciencias de la Vida, Universidad Andrés Bello, Santiago 8320000, Chile; anapimo@yahoo.es; 8 Genomics and Resistance Microbes (GeRM) Lab, Facultad de Medicina CAS—UDD, Instituto de Ciencias e Innovación en Medicina (ICIM), Santiago 7550000, Chile; 9 Instituto Agroalimentario de Aragón-IA2, Universidad de Zaragoza-CITA, Miguel Servet 177, 50013 Zaragoza, Spain; 10 Fundación ARAID, Avda. de Ranillas, 50018 Zaragoza, Spain

**Keywords:** antimicrobial resistance, *bla*_CTX-M_, Chile, domestic animals, *E. coli*, extended-spectrum beta-lactamases, wildlife

## Abstract

Antibiotic-resistant bacteria of critical importance for global health such as extended-spectrum beta-lactamases-producing (ESBL)-*Escherichia coli* have been detected in livestock, dogs, and wildlife worldwide. However, the dynamics of ESBL-*E. coli* between these animals remains poorly understood, particularly in small-scale farms of low and middle-income countries where contact between species can be frequent. We compared the prevalence of fecal carriage of ESBL-*E. coli* among 332 livestock (207 cows, 15 pigs, 60 horses, 40 sheep, 6 goats, 4 chickens), 82 dogs, and wildlife including 131 European rabbits, 30 rodents, and 12 Andean foxes sharing territory in peri-urban localities of central Chile. The prevalence was lower in livestock (3.0%) and wildlife (0.5%) compared to dogs (24%). Among 47 ESBL-*E. coli* isolates recovered, CTX-M-group 1 was the main ESBL genotype identified, followed by CTX-M-groups 2, 9, 8, and 25. ERIC-PCR showed no cluster of *E. coli* clones by either host species nor locality. To our knowledge, this is the first report of ESBL-*E. coli* among sheep, cattle, dogs, and rodents of Chile, confirming their fecal carriage among domestic and wild animals in small-scale farms. The high prevalence of ESBL-*E. coli* in dogs encourages further investigation on their role as potential reservoirs of this bacteria in agricultural settings.

## 1. Introduction

The current increase of antimicrobial resistance (AMR) is considered a main global threat to human and animal health [1,2]. AMR is responsible for thousands of human fatalities annually [3] and large economic losses that could reduce global GDP in 1–4% by 2050 [2,4]. The intense use of antibiotics in livestock production and humans is the main cause of the emergence and rapid spread of AMR [2,5]. In the last decade, the global growth of livestock has been associated with an increase in antibiotics use [2]. For example, 70% of antibiotics used in human medicine are consumed by animal production in the USA [6,7]. Extended-spectrum beta-lactamase-producing *Escherichia coli* (ESBL-*E. coli*) represent one of the highest burdens of AMR to public health and have globally spread in both hospital settings and the community [8]. ESBL-*E. coli* are commonly isolated from domestic animals such as cattle and dogs, but also wild animals [9,10,11,12]. Similar to humans, the misuse of third-generation cephalosporins in livestock generated a selective pressure resulting in the emergence and spread of ESBL-*E. coli* in this sector [9,13]. In contrast, the presence of ESBL-*E. coli* in wildlife is assumed to result from contamination in human-dominated environments [10,12,14].

The circulation of ESBL-*E. coli* across different animal populations requires an integrated One Health approach to better understand, predict, and prevent their dissemination [15]. However, most studies on ESBL-*E. coli* have focused on either one population (e.g., domestic or wild animals) or a large spatial scale (e.g., across cities or countries) [16,17,18,19]. For example, ESBL-*E. coli* have been detected worldwide in several livestock settings [13,20,21,22]. Likewise, ESBL-producing *Enterobacterales* have been found in at least 80 wildlife species since 2006 including rodents, bats, foxes, and wild birds [23,24,25,26]. Livestock or human proximity are often suggested as drivers of ESBL-*E. coli* in wildlife but, to our knowledge, no study has proven transmission from humans to wild animals [10,14,23]. Dogs living on farms could also contribute to the spread of ESBL-*E. coli* among agricultural settings because contact with livestock has been associated with an increased probability of ESBL-*E. coli* fecal carriage in dogs [27,28,29,30]. However, the circulation of ESBL-*E. coli* at the livestock and wildlife interface is still poorly understood [12,31,32].

Few studies on the circulation of ESBL-*E. coli* at the livestock and wildlife interface have been conducted in low- and middle-income countries (LMICs) [10,33,34,35,36]. Paradoxically, the consequences of AMR can be exacerbated in these countries by a higher number of bacterial infections and limited access to health facilities providing the appropriate antibiotic treatment [37,38]. Surveillance of AMR in livestock has been recommended by the World Health Organization (WHO), the Food and Agriculture Organization of the United Nations (FAO), and the World Organisation for Animal Health (OIE), but remains limited in LMICs [1,2,33]. Surveillance of AMR in wildlife and dogs is also mostly inexistent in LMICs. In this study, we use a One Health approach to compare the prevalence of ESBL-*E. coli* fecal carriage among livestock, dogs, and wild mammals located in small-scale agricultural settings of central Chile.

Chile, considered a high income economy but with an agricultural production more similar to LMICs, launched the ‘National plan to combat antimicrobial resistance’ in 2017, but no national surveillance has been implemented yet in the agricultural sector. ESBL-*E. coli* have not been detected in Chilean cattle herds [39,40], but have been isolated in feces from dogs [41], owls in rehabilitation centers [42], wild Andean condors (*Vultur gryphus*) [43] and gulls (*Leucophaeus pipixcan*) [36]. To our knowledge, no study has investigated the ESBL-*E. coli* fecal carriage of livestock nor simultaneously focused on dogs and wild mammals living closely to livestock. Central Chile hosts a large diversity of endemic terrestrial mammals including foxes and rodents [44,45] but also invasive species such as the European rabbit (*Oryctolagus cuniculus*) that has colonized most of the country [46,47,48]. Rodents and rabbits are commonly found living on farms and interacting with dogs and livestock [49,50]. Similarly, 85% of the territory of the Andean fox (*Lycalopex culpaeus*) overlaps with human-dominated habitat in central Chile [51]. This creates the potential for fecal-oral and environmental bacterial transmission between livestock and wild animals, which remains largely unknown. Previous studies focusing on foxes in the central region have identified the presence of *bla*_CTX-M_ genes, but the bacteria carrying the gene was unknown [52]. The aims of this study were (i) to estimate and compare the prevalence of ESBL-*E. coli* fecal carriage between livestock, dogs, and wild mammals living in the same agricultural setting of central Chile, (ii) to detect the presence of the most common ESBL genes including *bla*_CTX-M_, *bla*_TEM_, and *bla*_SHV_, and (iii) use high resolution molecular typing to assess potential ESBL-*E. coli* transmission within farms or between different species.

## 2. Materials and Methods

### 2.1. Sample Collection

**Figure 1:**
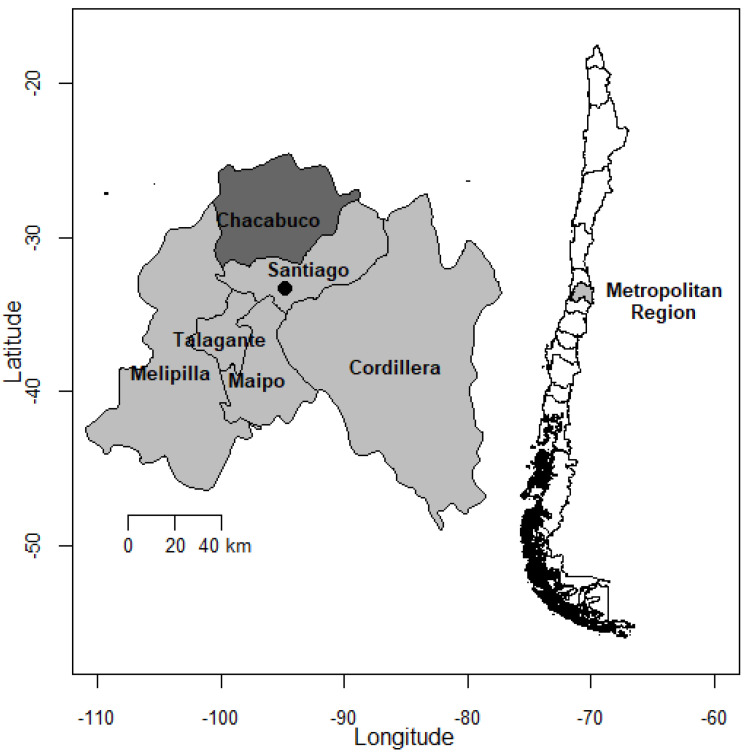
Study area. The inset figure shows the Chacabuco province within the Metropolitan region where farms and wildlife were sampled. Exact farm locations are not given to maintain our confidentiality agreement with farmers. Maps were obtained from the GADM (http://www.gadm.org//, accessed on 15 April 2021) database using the *getData* function from the *raster* package of R.

Fresh fecal samples were collected between March 2019 and September 2019 from livestock, dogs, and wildlife in and around 13 farming localities located in the municipalities of Colina (33.1045° S, 70.6159° W) and Lampa (33.2827° S, 70.8793° W) of the Chacabuco province in the Metropolitan Region of central Chile, in the peri-urban area of the Santiago Capital City (Figure 1). A farming locality was either a single private farm or an area where livestock from different owners grazed together and received the same health treatments. The province of Chacabuco includes mainly small- to medium-scale farmers, with an estimated livestock population of 10,662 cattle (mean: 38 animals/farm), 45,821 pigs (587/farm), 5490 goats (59/farm), 4441 sheep (42/farm), and 2897 horses (4/farm) [53]. Farms were randomly selected from a list provided by the Municipality’s agrarian unit, accounting for areas overlapping with the known territory of wildlife as previously described [52]. Our sampling focused mainly on cattle because they had the highest potential of overlapping with wild mammals since they often free-ranged within wildlife habitat during our study period.

We focused on sampling the most common wild mammals encountered in those farms including several species of endemic and invasive rodents, the invasive European wild rabbit and the Andean fox, who predates these herbivore species [54,55]. These species were previously determined by discussions with farmers and the municipality’s agrarian unit during preliminary visits to the farms. Peri-urban and wild rodents were live captured, sampled, and released using Sherman traps. Fifty traps were placed in and around each sampled farm for at least 4 consecutive days and checked for captured rodents daily. Rectal swabs were collected from alive individuals immobilized, using gloves and protective equipment. Rodents were identified at the genus or species level based on morphological characteristics. Fresh fecal samples from European rabbits were collected early in the morning by identifying rabbit dens in areas where farmers commonly observed rabbits. To avoid sampling the same individual twice, we only collected fresh sample feces from the same den if they were more than 4 m apart, and only sampled each den once. Fresh fecal samples from foxes were collected by walking known paths where foxes were previously captured in the area [56]. Fresh samples from foxes were identified and differentiated from dog feces by their distinct ‘fruit’ seeds and morphology contained on the sample. To avoid sampling the same individual twice, we only collected a fresh sample in localities that were more than 5 km apart, considering 5 km^2^ as the average home range size of foxes in this area [52]. Dogs were sampled by directly taking rectal swabs or waiting until the dog defecated, depending on whether the owner considered that the dog could be aggressive or not during sampling. For all samples taken from the ground, we only collected the portion that was not in contact with the ground to avoid bacterial contamination from the soil. This study was approved by the Ethical Committee of the Universidad Andrés Bello (permit number: 018/2018). The capture and sampling of rodents were also approved by the Servicio Agricola Ganadero (permit number: 2118/2019).

### 2.2. Sample Size and Prevalence Estimation

The required sample size needed to estimate the prevalence of ESBL-*E. coli* in livestock (defined as the number of animals harboring at least one isolate of ESBL-*E. coli* over the total number of sampled animals) was calculated with the program Epi Info 7.2.2.6^TM^ [57]. To our knowledge, no previous study has estimated the prevalence of fecal carriage of ESBL-*E. coli* among livestock in Chile. Thus, we assumed an expected prevalence of ESBL-*E. coli* of 30%, similar to a study conducted around the Lima capital in Peru with similar farm characteristics [12]. Based on this expected prevalence, a margin of acceptable error of 5% and a confidence interval of 95%, the minimum number of livestock to be sampled in the region was 323.

Based on previous studies on wildlife and dogs, we assumed an expected prevalence of 5% to estimate our sample size. In fact, 5% prevalence of ESLB-*E. coli* was found in wild rodents in China [34,58], no bacteria were found in a previous study conducted in European wild rabbit in Portugal [59], 4% prevalence was found in wild foxes of Portugal [60], and 8% was found in the only study conducted on dogs in Chile [41]. Based on an expected ESBL-*E. coli* prevalence of 5%, a margin of acceptable error of 5% and a confidence interval of 95%, the minimum number of animals to be sampled was 73. We aimed to collect 73 samples per wildlife group (e.g., foxes, rabbits, and rodents). However, giving the intrinsic lower density of foxes compared to small mammals and logistic constraints for finding foxes, we expected a much lower sample size for this species.

### 2.3. Microbiology Analyses

Fresh fecal samples were collected using Stuart Transport Medium (Deltalab^®^) and cultured within 3 days of sampling. Swabs were screened for cefotaxime non-susceptible *E. coli* by direct incubation in standard atmospheric conditions (100 kPa) at 37 °C for 24 h in a MacConkey medium containing 2 µg/mL of cefotaxime sodium salt (Sigma-Aldrich, St. Louis, MO, USA) [61]. Up to 3 isolates with different morphotypes compatible with *E. coli* per sample/plate were purified and then stored at −80 °C for further analyses. Bacterial species were confirmed by matrix-assisted laser desorption ionization-time of flight (MALDI-TOF) mass spectrometry (BioMérieux, Marcy l’Etoile, France) at the Genomics and Resistant Microbes (GeRM) Group of the Millennium Initiative for Collaborative Research on Bacterial Resistance (MICROB-R).

Cefotaxime non-susceptible *E. coli* isolates indicating ESBL were tested for antimicrobial susceptibility to 8 antibiotics from 6 classes including chloramphenicol (phenicol), ciprofloxacin (quinolone), sulfamethoxazole (sulfonamide), amikacin (aminoglycoside), tobramycin (aminoglycoside), ertapenem (carbapenem), tetracycline, and gentamicin (aminoglycoside). Multidrug resistance (MDR) was defined as resistance to at least 1 agent of 3 or more antibiotic classes [62]. The *E. coli* ATCC25922 strain was used for quality control and clinical breakpoints were in accordance with CLSI M100:28ED recommendations [61].

Extended-spectrum beta-lactamase production was confirmed in all cefotaxime non-susceptible *E. coli* isolates by the double-disk synergy test [30] on Müller Hinton agar (Difco, BD, Sparks, MD, USA) with and without the AmpC inhibitor phenylboronic acid (Sigma-Aldrich). Briefly, disks of ceftriaxone (30 µg), ceftazidime (30 µg), cefepime (30 µg), and aztreonam (30 µg) were used along with a disk of amoxicillin with clavulanic acid (30 µg) placed in the center of the plate at approximately 20 mm. Inhibition zones (ghost zones) observed around any of the cephalosporin disks towards the disk containing the clavulanic acid after 18–20 h of incubation at 37 °C aerobically were considered as a positive result to produce ESBL.

The presence of the most common ESBL-encoding genes in *E. coli* isolates including *bla*_CTX-M_, *bla*_TEM_, and *bla*_SHV_, was tested by a previously described multiplex PCR [63]. DNA samples from reference *bla*_CTX-M_, *bla*_TEM_, and *bla*_SHV_ strains stored at the Universidad de Concepción’s Laboratory of Research in Antimicrobial Agents were used as positive PCR controls. The specific group of each CTX-M alleles (CTX-M groups 1, 2, 8, 9, and 25) were detected by multiplex-PCR as described previously [64]. In order to explore the phylogenetic relationships between ESBL-*E. coli* isolates within and between host species or localities, isolates were fingerprinted by ERIC-PCR according to Bilung et al. [65].

### 2.4. Statistical Analyses

The prevalence of ESBL-*E. coli* was reported and 95% confidence intervals were calculated using the *binom.confint* function (Agresti-Coull method) in the *binom* package in R 3.6.1 [66]. Significant differences in prevalence between populations were tested using the Fisher’s exact test in R, since the limited number of observations prevented the use of a Chi-Squared test. We constructed a dendrogram based on the ERIC-PCR electrophoretic patterns using the BioNumerics software v8.0 (Applied Maths, Belgium) and R [65,66]. An UMPGA dendrogram was built based on scaled densitometry curves from the ERIC-PCR obtained from BioNumeric using the *hclust* function of the *dendextended* R package.

## 3. Results

**Figure 2:**
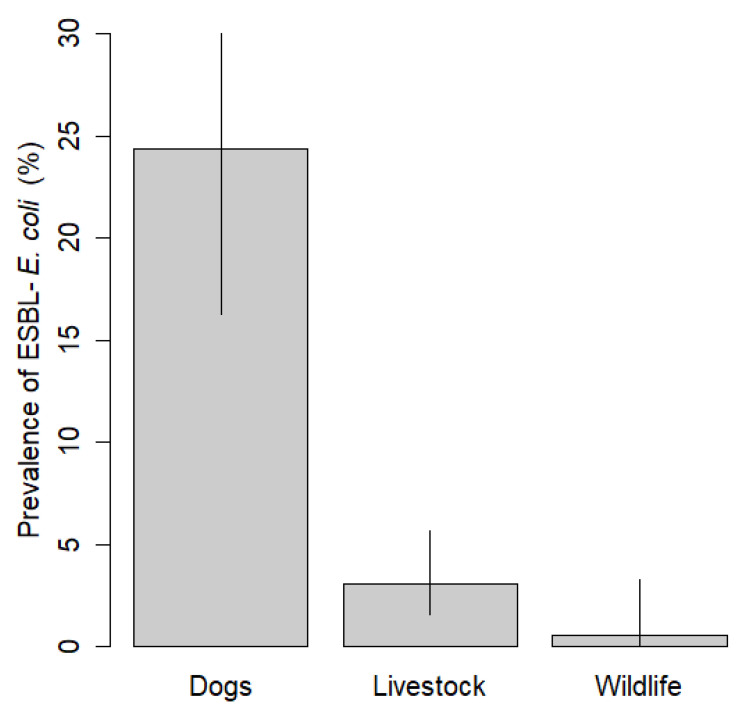
Prevalence of ESBL-*E. coli* per species in small-scale farms of central Chile; 95% confidence intervals were estimated using the *binom.confint* function (Agresti-Coull method) in the *binom* package in R.

ESBL-*E. coli* fecal carriage was detected in chickens, cattle, pigs, sheep, goats, dogs, and one wild rodent (*Octodon degus*). The prevalence of ESBL-*E. coli* fecal carriage was significantly higher among dogs (24% [CI: 16–35%]; 20 out of 82) compared to livestock (3% [CI: 2–6%]; 10 out of 324, Fisher’s exact test, Odds Ratio (OR) = 10.0, *p* < 0.0001) and wildlife (0.5% [CI: 0–3%]; 1 out of 186, Fisher’s exact test, OR = 58.8, *p* < 0.0001) (Figure 2). The prevalence of ESBL-*E. coli* in livestock was also significantly higher than the prevalence in wildlife (Fisher’s exact test, OR = 25.4, *p* < 0.0001). At least 1 animal carrying ESBL-*E. coli* was detected in 7 out of the 13 (54%) farm localities sampled. In all 3 farms where livestock carried ESBL-*E. coli* and dogs were sampled, at least 1 dog also carried ESBL-*E. coli*. Likewise, the wild rodent carrying ESBL-*E. coli* was detected in a farm where one cow also carried ESBL-*E. coli*.

**Figure 3:**
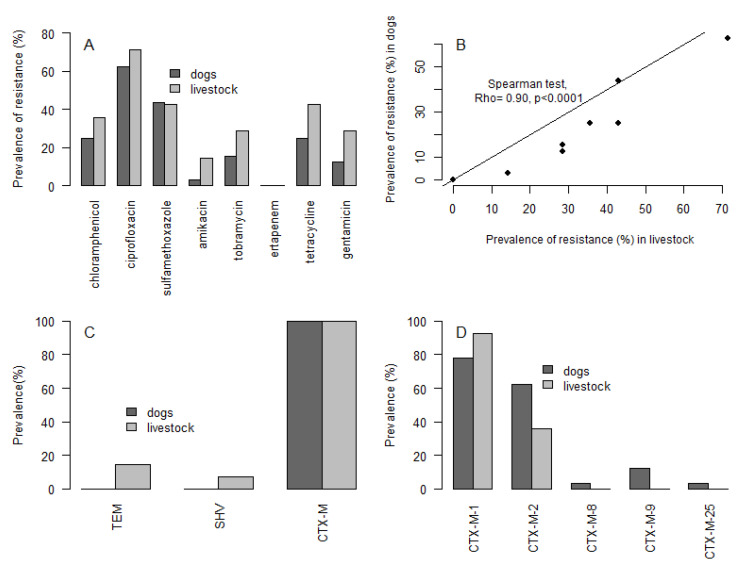
(**A**) Prevalence of resistance to other antibiotic families among ESBL-*E. coli* isolates in dogs and livestock; (**B**) Correlation of the prevalence of resistance to each antibiotic between livestock and dogs; (**C**) Prevalence of *bla*_TEM_, *bla*_SHV_, and *bla*_CTX-M_ in ESBL-*E. coli* isolated from livestock and dogs; (**D**) Prevalence of CTX-M groups identified in ESBL-*E. coli* isolates from livestock and dogs.

A total of 47 ESBL-*E. coli* isolates (confirmed by the double-disk synergy test) from 33 animals were analyzed. Fourteen ESBL-*E. coli* isolates were obtained from 10 livestock, 32 isolates from dogs and 1 isolate from a mouse. ESBL-*E. coli* isolates from livestock were resistant to a median (mean) of 1 (2.6) (range: 0–6) out of 8 antibiotics tested, while ESBL-*E. coli* isolates from dogs were resistant to a median (mean) of 1 antibiotic (1.9) (range: 0–6) (Figure 3A). Overall, 21% of ESBL-*E. coli* isolates from livestock and 31% from dogs were susceptible to all antibiotics, 36% of ESBL-*E. coli* isolates from livestock and 21% from dogs were resistant to one antibiotic, and 43% of ESBL-*E. coli* isolates from livestock and 48% from dogs were resistant to two or more antibiotics. Additionally, 43% of ESBL-*E. coli* isolates from livestock, 47% from dogs and an isolate from one rodent were multidrug resistant (MDR). The ESBL-*E. coli* isolated from a rodent sample was resistant to chloramphenicol, sulfamethoxazole, and ciprofloxacin. More than 20% of ESBL isolates were resistant to ciprofloxacin, chloramphenicol, sulfamethoxazole, and tetracycline in both dogs and livestock. In contrast, no resistance was observed against ertapenem. Among ESBL isolates, the prevalence of resistance to each antibiotic was highly correlated between livestock and dogs (Spearman’s test, Rho = 0.90, *p* < 0.0001), but livestock had a slightly higher prevalence than dogs for most antibiotics (Figure 3B).

ESBL-*E. coli* isolates from dogs were only encoded by the CTX-M genotype while all isolates from livestock carried CTX-M (100%), followed by TEM (14%), and SHV (7%) genotypes (Figure 3C). Among the most common CTX-M groups searched, 93% of ESBL-*E. coli* from livestock carried *bla*_CTX-M-group 1_ and 36% carried *bla*_CTX-M-group 2_ genes (Figure 3D). Isolates from dogs carried a more diverse pool of CTX-M genotypes with 78% carrying CTX-M from group 1, followed by group 2 (63%), group 9 (12.5%), group 8 (3%, one isolate), and group 25 (3%). The ESBL-*E. coli* isolate found on a wild mouse carried CTX-M from group 1.

**Figure 4:**
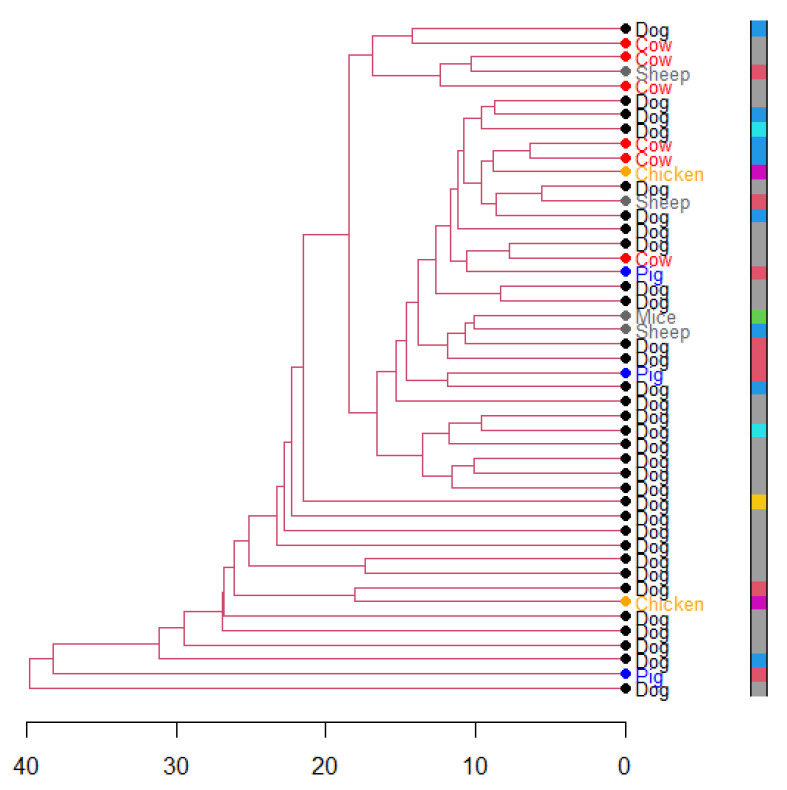
Dendrogram produced by the analysis of the ERIC-PCR of ESBL-*E. coli* isolates from livestock and dogs using the UMPGA method in R. The colored column on the right side represents different farm localities where isolates were recovered.

The dendrogram analysis of the ERIC-PCR results showed a high diversity of ESBL-*E. coli* clones within species and farm localities. No visual clustering by species nor farm localities was observed (Figure 4). However, ESBL-*E. coli* isolates from a cow and a dog from the same farm locality clustered together.

## 4. Discussion

The spread of AMR at the interface between domestic animals and wildlife remains poorly understood, particularly in low-income rural areas without specific barriers to limit the interaction between domestic and wild animals. In this study, we simultaneously estimated the prevalence of ESBL-*E. coli* fecal carriage among livestock, dogs, and wild mammals among small-scale agricultural localities of central Chile. The prevalence of ESBL-*E. coli* fecal carriage was lower in livestock (3%) and wildlife (less than 1%) compared to dogs (24%), suggesting that dogs can be an important carrier of these bacteria in agricultural settings. Dogs carried ESBL-*E. coli* in the three farms where ESBL-*E. coli* were detected in livestock, highlighting the potential sharing of these bacteria between dogs and livestock. Among ESBL-*E. coli* isolates, five CTX-M groups including groups 1, 2, 8, 9, and 25 were detected, with most isolates carrying CTX-M group 1. Molecular typing of ESBL-*E. coli* by ERIC-PCR showed no cluster of isolates by neither species nor locality, suggesting a wide range of ESBL-*E. coli* strains circulating on agricultural settings and highlighting the potential for cross-species transmission of either bacteria or antibiotic resistance genes.

ESBL-*E. coli* have been detected across livestock in South America, with prevalence in cattle ranging from 18% in Brazil to 48% in Peru [12,67]. In this study, we detected ESBL-*E. coli* fecal carriage in cattle, swine, sheep, and chicken, showing the widespread dissemination of these bacteria in agricultural settings. This is the first report of ESBL-*E. coli* in cattle in Chile, although their prevalence was low (3%) compared to a similar study in Peru estimating a prevalence of 48% among small-scale farmers in the Lima region [12]. The observed prevalence in Chile is similar to farms in high-income countries such as France or Denmark, where the restriction of third-generation cephalosporins has been associated with a reduction in ESBL-*E. coli* [68,69]. The high prevalence of resistance to ciprofloxacin (over 60%) found in ESBL-*E. coli* isolated from domestic animals in this study is consistent with the high level of plasmid-mediated quinolone resistant found in 74% of ESBL-*E. coli* isolated from Chilean hospitals [70] and a high prevalence of resistance to ciprofloxacin (84%) in ESBL-*E. coli* recovered from intensive care units of Southern Chile [71]. The presence of ESBL-*E. coli* could result from low but existing selective pressure by the use of third generation cephalosporins in these farms, which requires further investigation. In a similar agricultural setting of Peru, the low use of cephalosporins [72] was associated to a high prevalence of ESBL-*E. coli* in livestock (50%) [12], suggesting that factors other than antibiotic use can influence AMR. For example, farm hygiene, herd size, contact with humans or other husbandry conditions such as storage of slurry in a pit have been associated with the presence of ESBL-*E. coli* in livestock [13,20,21].

The low prevalence of ESBL-*E. coli* in wildlife (less than 1%) is similar to other studies focusing on ESBL-*E. coli* among wildlife in Latin America and other LMICs [12,73]. For example, a previous study estimated a 4% prevalence of ESBL-*E. coli* among vampire bats (*Desmodus rotundus*) in Peru using a similar methodology for screening [12]. Previous studies conducted in Chile and Latin America have detected the presence of ESBL-*E. coli* on wild birds including gulls [36], Andean condors [43], and three species of owls [42]. Likewise, *bla*_CTX-M_ genes have been previously detected using qPCR methods from feces in Andean foxes [52] and the guiña (*Leopardus guigna*) [74], although the bacteria species carrying the genes, and whether it was expressed or not, remains unknown. To our knowledge, this is the first study to report *E. coli* carrying CTX-M group 1 on wild mammals in Chile. The origin of ESBL-*E. coli* found in a rodent remains to be clarified. Given the presence of similar *bla*_CTX-M_ genes among a nearby farm and a wide variety of ESBL-*E. coli* strains circulating, one potential explanation is the transmission of *bla*_CTX-M_ from domestic animals, although other potential contamination sources (e.g., humans, water contamination) cannot be discarded.

The high prevalence of ESBL-*E. coli* found in dogs (24%) highlights their role as either passive ‘receivers’ or reservoirs of ESBL-*E. coli* in agricultural settings. Although there are only a limited number of studies estimating the prevalence of ESBL-*E. coli* among dogs, previous studies have shown a prevalence in Latin American dogs ranging from 9–30%, and a global prevalence of 7% [30,75,76,77,78,79]. The detection of ESBL-*E. coli* in dogs has been associated with previous antibiotic treatment, but also close contact with livestock, implying the potential transmission of these bacteria between livestock and dogs [29,30,80]. The latest is also suggested by our study, as the three farms where we detected ESBL-*E. coli* in livestock also had a dog carrying ESBL-*E. coli*. Molecular typing by ERIC-PCR showed no cluster of ESBL-*E. coli* by host species, while isolates sampled from a cow and a dog at the same farm clustered together. These results suggest that bacterial strains or ESBL genes such as *bla*_CTX-M_ could be exchanged between host populations. Overall, the circulation of ESBL-*E. coli* among dogs highlights the potential public health risk for domestic animals but also for dog owners, given the potential spillover of bacteria from dogs to humans [28,29,81]. Moreover, the higher prevalence observed in dogs compared to livestock suggests that ESBL-*E. coli* could be spreading from dogs to livestock, and not necessarily in the other direction, as most previous studies have assumed.

Our study constitutes one of the first One Health approaches to simultaneously address the circulation of ESBL-*E. coli* among livestock, dogs, and wildlife in a rural setting. However, several future research can complement our findings and provide further insight into the selection and spread of AMR among these compartments. First, the limited sample size of foxes prevented a more accurate estimation of ESBL-*E. coli* prevalence in this species. Thus, we could not conclude whether predators or preys are more likely to carry ESBL-*E. coli* in this setting. Secondly, the low selective pressure for ESBL-*E. coli* should be confirmed by studies on antibiotic use among farmers in these agricultural settings [72], which are currently lacking in Chile. Although the use of antibiotics in Chilean terrestrial livestock remains unknown, the national health authority (Servicio Agricola Ganadero) advises the use of fluroquinolones and cephalosporins as a last resource antibiotic in livestock, following a susceptibility test [82]. Antibiotic residues of tetracyclines, beta-lactams, aminoglycosides, and macrolides have been found in eggs from backyard poultry production [83]. Thirdly, although the ERIC-PCR technique used has a high resolution and allows us to differentiate among *E. coli* strains from the same locality and host species [65], several other molecular techniques can improve our understanding of the transmission dynamics of resistance genes and *E. coli*. For example, future work could determine the pathogenic potential of these strains using whole genome sequencing, or whether *bla*_CTX-M_ genes are carried by specific mobile elements such as plasmids. Finally, future research should identify associated factors to ESBL-*E. coli* fecal carriage in each animal population (e.g., individual characteristics of dogs and cattle).

## Data Availability

The data presented in this study are available within this article.
